# Collagen and platelet-rich plasma in partial-thickness rotator cuff injuries. Friends or only indifferent neighbours? Randomised controlled trial

**DOI:** 10.1186/s12891-022-06089-9

**Published:** 2022-12-20

**Authors:** Piotr Godek, Beata Szczepanowska-Wolowiec, Dominik Golicki

**Affiliations:** 1 Sutherland Medical Center, 04-036 Warsaw, Poland; 2 Institute of Health Sciences, Collegium Medicum, The Jan Kochanowski University, Kielce, Poland; 3 Department of Experimental and Clinical Pharmacology, Medical University of Warsaw, Warsaw, Poland

**Keywords:** Collagen, Conservative treatment, Platelet-rich plasma, Rotator cuff, Tendinopathy

## Abstract

**Background:**

Partial-thickness rotator cuff injuries (PTRCI) are the sum of degenerative, overload, and microtrauma processes. An external supply of collagen and platelet-rich plasma (PRP) could potentially counteract the deterioration of degenerative tendinopathy. This study aimed to compare the effectiveness of collagen with PRP, PRP alone, and collagen alone in the treatment of PTRCI.

**Methods:**

Ninety patients with PTRCI were randomised and treated with ultrasound-guided injections into the shoulder bursa every consecutive week: Group A – collagen with PRP (*n* = 30), Group B – collagen alone (*n* = 30), and Group C – PRP alone (*n* = 30). Primary outcomes were pain intensity measured in control points on a numeric rating scale (NRS), QuickDash, and EQ-5D-5L questionnaires at the initial assessment (IA) and control assessments after 6 (T1), 12 (T2), and 24 (T3) weeks, respectively.

**Results:**

No statistical differences were found between groups in primary outcomes, although there was a trend towards improvement in Groups A and C (opposite to Group B) between T2 and T3. The following parameters were also observed: rotator cuff discontinuity (*n* = 3, one case in each group) and rotator cuff regeneration (*n* = 22 in Group A, *n* = 20 in Group B, and *n* = 23 in Group C).

**Conclusions:**

Combined therapy of collagen and PRP in PTRCI presents similar effectiveness to monotherapies with collagen or PRP.

**Trial registration:**

The study was prospectively registered on the NCT Trial Center (identification number: NCT04492748) on 30.07.2020.

## Background

Rotator cuff injuries (RCI) rank third in the population prevalence among musculoskeletal system pathologies (16%) after lumbar spine pain (25%) and knee pain (19%). The prevalence of RCI is 5–39%. It increases with age, reaching over 30% in patients over 60 years old, with a great majority described as rotator cuff tendinopathy (RCT), mainly in the form of partial-thickness RCI (PTRCI) as an emanation of degenerative changes [1]. Over 85% of the dry mass of the rotator cuff tendons is type I collagen. Therefore, disorganisation of collagen fibres and a negative metabolic balance of collagen underlie the macroscopic lesions visible in ultrasound or magnetic resonance imaging (MRI) [2].

Most often, the PTRCI concerns the supraspinatus tendon (SSP), which is a crucial factor in centring the humeral head during upper limb elevation [3, 4]. In traumatic cases, the subscapularis and infraspinatus tendons are often affected by long head biceps instability [5]. There are several reasons for the degenerative process leading initially to oedema, micro-perforations, and then full-thickness tendon lesions: 1) age-related weakening of blood supply near the SSP insertion, 2) concomitant degenerative spurs of acromioclavicular joint or acromion shape as a direct cause of the subacromial impingement, 3) disturbed muscle timing between rotator cuff (RC) and deltoid usually associated with cervical spondylosis, (scapular dyskinesis), 4) shoulder joint multidirectional instability as a result of capsule-ligamentous elements laxity and disturbed contact of joint surfaces with RC posterior impingement [6, 7].

The consequences of RCI include scapular dyskinesis and upper and anterior migration of the humeral head, followed by subacromial bursitis [8]. The PTRCI results in shoulder pain radiating to the elbow, muscular weakness, impaired function, and disability [9]. Medium- to long-term clinical and functional outcomes of isolated and combined subscapularis tears can be repaired arthroscopically [10]. Due to the risk of surgery, reduced strength of RC tendons, and a significant risk of injury recurrence, conservative options are the first choice, especially in older adults [11, 12]. These conservative procedures comprise physical therapy and pharmacotherapy with steroid injections for anti-inflammatory effects, platelet-rich plasma (PRP), collagen injections, and autologous conditioned serum (ACS) for regenerative effects, as well as rehabilitation management [13–15].

PRP has well-evidenced effects in alleviating symptoms and slowing tendon degeneration, demonstrating its advantage over steroid administration or prolotherapy [16–18]. Collagen injections in the vicinity of the injured tissue (into the tendon itself or into the subacromial bursa) also have a suppressing effect on the negative balance of collagen metabolism. The premises for this type of injection are reports showing a reduction in pain after collagen injections compared to steroid injections and a significant acceleration of the proliferation and migration of tenocytes cultured in an exogenous collagen environment in vitro [19, 20]. The same is true for the synergic effects of collagen and PRP confirmed in multiple studies that utilised tendon-like cell models, in which increased cell proliferation was observed with the addition of various PRP products. This suggests that PRP products positively affect the cell’s mitogenic activity, collagen production, and the optimisation of the collagen I/III ratio [21].

These positive effects and their consequences for clinical significance have not yet been demonstrated, and this gap was our main incentive for initiating a comparative study. The present study aimed to compare the effectiveness of three treatment concepts—collagen with PRP (combined therapy), collagen alone, and PRP alone as monotherapy—in the treatment of PTRCI.

## Methods

### Study design and participants

The study design was a single-centre open randomised controlled trial. All data were collected at Sutherland Medical Center (SMC) (Warsaw, Poland). Three groups of patients, each including 30 participants, were enrolled in the study. Patients who met the inclusion criteria were allocated randomly according to the computer-generated randomisation list (block randomisation; block size = 6). No changes in allocation or the study’s methodology took place throughout the study.

### Ethical considerations

The trial protocol was approved by the Bioethics Committee at the Faculty of Health Sciences of Jan Kochanowski University in Kielce (approval no.: 15/2020, approval date: 18.05.2020). All experiments were performed in accordance with and following the Declaration of Helsinki principles and Good Clinical Practice. Written informed consent was obtained from all participants prior to the injections and the publication of their individual data. The study was performed at SMC in Warsaw, Poland. The trial was registered on Clinicaltrials.gov (NCT04492748) on 30.07.2020 (Initial Release), last updated on 03.10.2021. Unique Protocol ID: SMC2020001. Brief Title: Rotator Cuff Tendinopathy Conservative Treatment with Collagen, PRP or Both (RCCT).

### Qualification criteria

Inclusion criteria were: (1) clinical signs and symptoms of rotator cuff pathology, (2) an adult person consenting to injections, (3) partial-thickness rotator cuff injury confirmed by ultrasound examination without coexisting severe pathologies (systemic inflammatory disease, malignancy, severe stage of osteoarthritis), (4) no traumatic event, and (5) written informed consent to participate in the study. Exclusion criteria were: (1) full-thickness rotator cuff injury, (2) acute, traumatic injuries requiring surgical treatment, (3) coexisting injuries of the shoulder joint requiring other intervention, (4) severe pathologies of the shoulder of another origin (systemic inflammatory disease, malignancy, severe stage of osteoarthritis), and (5) lack of consent to participate in the study.

### Recruitment procedure

**Fig. 1:**
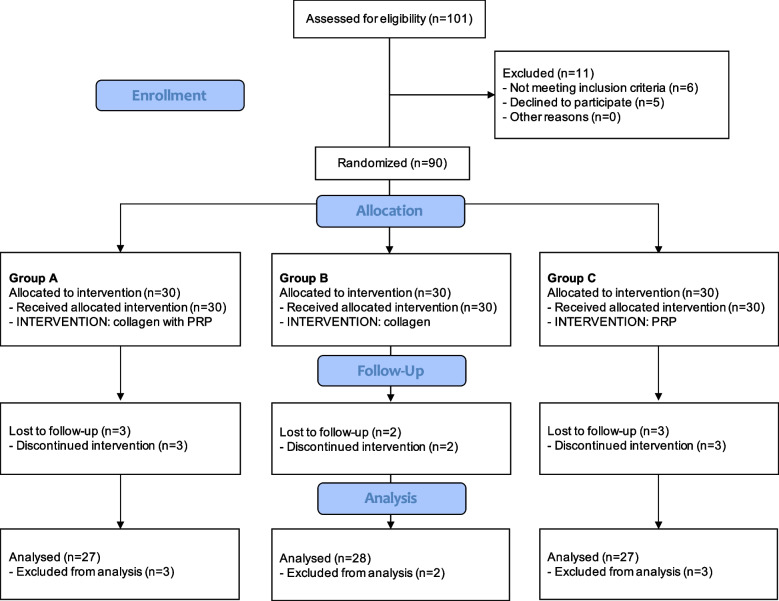
CONSORT flow chart of study participants. *Abbreviations: n* number of participants, *PRP* platelet-rich plasma

A group of 101 patients were screened for eligibility, of which 90 met the inclusion criteria and were randomised between 15.06.2020 and 19.11.2020 into three comparative groups: A (*n* = 30; combined collagen with PRP), B (*n* = 30; collagen alone), and C (*n* = 30; PRP alone). Eight patients who were lost to follow-up assessment did not obtain the T3 control due to their discontinuation of intervention. One person from Group A did not finish the therapy for reasons other than therapy intolerance and had no T1, T2, or T3 observations. Two persons, one in each of Groups A and C, quit the study after T1. One person from Group C left the follow-up appointment after T2 due to a lack of improvement and asked for a change in therapy. Two patients, one in each of Groups A and C, experienced total RC tears before the end of the observation—between T2 and T3. Further, two patients from Group B were found to have had complete RC injury at the T3 visit. The follow-up process reached 28.05.2021. The recruitment and follow-up processes according to CONSORT guidelines are presented in Fig. 1.

### Study outcomes

Primary outcomes included pain intensity measured by the numeric rating scale (NRS, 0–10; 0 – no pain, 10 – maximal pain), QuickDash questionnaire (0–50; 0 – no disability, 50 – maximal disability), EQ-5D-5L questionnaire (five dimensions: MO – mobility, SC – self-care, UA –usual activities, PD – pain and discomfort, AD – anxiety, and depression; each dimension with five levels of limitations: 1 – no limitation, 5 – maximal limitation; visual analogue scale EQ-VAS 0–100; 0 – the worst health status, 100 – optimal health status). Follow-up schedule for primary outcomes: Initial assessment (IA), 6 (T1), 12 (T3), and 24 (T3) weeks after the last injection. Secondary outcomes included the percentage of patients in each group where the RC continuity was preserved with the desired evolution of RC cross-section width and the percentage of patients with US-related signs of RC regeneration. In addition, secondary outcomes were assessed at IA and 24 (T3) weeks after the last injection.

### Outcome measures

During the IA, patients were asked to evaluate the intensity of the pain (NRS, ranging from 0 – no pain to 10 – extreme pain) and to complete widely used, validated questionnaires: QuickDash (0–50) using 11 items to measure physical function and symptoms of the upper limb and the EQ-5D-5L (descriptive part – Utility Index and EQ-VAS 0–100) measuring general health status. Ultrasound examination of the shoulder was performed using an Alpinion E-CUBE 12 device (ALPINION MEDICAL SYSTEMS Co., Ltd., South Korea), linear transducer L3-12H (3–12 MHz). The SSP tendon width (cross-section in mm) was measured in the internal rotation position of the arm. We distinguished the following ultrasound patterns of PTRCI: bursa-sided (BS), joint-sided (JS), intra-tendon (IT), and oblique or focal (OF). The measurement in BS and JS types was performed at the narrowest point (the follow-up measure estimates the tendency for an increase in the RC width as a sign of regeneration). In the IT or OF type of injury, the measurement was performed at the thickest point of RC (follow-up estimated tendency for reduction of inflammatory and oedematous overgrowth of the RC as a sign of regeneration). It should be emphasised that ultrasound examination of the shoulder, especially RC thickness, which was performed according to the standards and based on the current state of the art, is highly sensitive and specific for the diagnosis of supraspinatus tears and presents equivalent capability to MRI in the diagnosis of both full- and partial-thickness RC tears (PT-RCTs) [22].

### Treatment protocol

Participants were randomised into three groups: Group A – collagen (3 vials of MD-Shoulder collagen – total 6 ccs) simultaneously with PRP GLOFINN (10 ccs whole blood, double centrifugate, leukocyte rich PRP, the volume of PRP – 2 ccs); Group B – collagen alone (3 vials of MD-Shoulder collagen); and Group C – PRP GLOFINN alone. Each group was treated with three US-guided injections into the subacromial bursa using the in-plane technique. Injections were performed every consecutive week by the same physician (PG). All patients were allowed to continue a rehabilitation protocol to preserve a safe, pain-free range of motion, postural exercises, and scapular stabilisation exercises. Any exercises with resistance that would compromise the healing process of the RC were prohibited.

### Sample size

The sample size analysis was calculated using Statistica 13 (TIBCO Software Inc. Palo Alto, USA). Sample size analysis was estimated based on Cohen’s statistics. The power of the test was set at 0.8 and the significance level at 0.05, assuming that the effect size was f = 0.35. This allowed us to establish that the research sample for the three compared groups should not be smaller than 90 subjects (each group with 30 participants).

### Statistical analysis

Statistica 13 (TIBCO Software Inc. Palo Alto, USA) and IBM SPSS v. 25 (IBM Corporation, New York, USA) were used for statistical analysis. For measurable variables, mean values (M), standard deviations (SD) and extreme values (Min-Max) were calculated. The frequency of their occurrence (percentage) was calculated for qualitative variables. In order to check the normality of the distribution of the examined variables, the Shapiro-Wilk test was used. The qualitative variables were compared between the groups using the chi-square test (χ2). The comparison of quantitative variables between groups was performed using a one-way ANOVA. Comparison of the results between groups and depending on the measurement time was performed using an ANOVA of repeated measures with post-hoc analysis (Tukey test). *P* < 0.05 was considered statistically significant. Calculation of differences in values between the baseline IA and the T3 point was also performed, and then the mean values of this difference were taken to compare primary outcomes between the groups.

## Results

The treatment was accomplished in 89 patients (99%). All check-up visits were passed by 91.1% of the patients. In 82 patients who completed therapy and obtained T3 control, three SSP total injuries were observed (3.6%). Statistically significant differences were found in the frequency of the RC tendinopathy phase (*p* = 0.031). However, there were no statistically significant differences between the groups, considering the other variables. A detailed demographic characteristic of the study group is shown in Table 1.

**Table 1:** Comparison of demographic characteristics of groups

Characteristic	Total	Group			*p*-value
		A	B	C	
Number of patients, n	90	30	30	30	–
Female, n (%)	42 (46.7)	9 (30.0)	18 (60.0)	15 (60.0)	0.060*
Age, years, mean M ± SD (range)	54.5 ± 14.7 (24–91)	49.6 ± 15.3 (24–91)	56.0 ± 15.5 (24–91)	55.8 ± 12.6 (36–79)	0.161**
Duration of complaints weeks, mean ± SD (range)	21.8 ± 28.5 (1–230)	17.5 ± 17.0 (2–52)	18.8 ± 15.9 (1–52)	29.2 ± 43.2 (3–230)	0.177**
RC tendinopathy phase, n (%):					0.031*
Acute phase patients,	10 (11.1)	7 (23.4)	2 (6.7)	1 (3.3)	
Subacute phase patients	27 (30.0)	10 (33.3)	11 (36.7)	6 (20.0)	
Chronic phase patients	53 (58.9)	13 (43.3)	17 (56.7)	23 (76.7)	
Injury type, n (%):					0.306*
Bursa Side	5 (5.6)	1 (3.3)	3 (10.0)	1 (3.3)	
Joint Side	49 (54.4)	21 (70.0)	12 (40.0)	16 (53.3)	
Intratendinous	22 (24.4)	6 (20.0)	8 (26.7)	8 (26.7)	
Oblique and focal	14 (15.6)	2 (6.7)	7 (23.3)	5 (16.7)	
Side of complaints, n (%):					0.731*
Right	49 (54.4)	16 (53.3)	18 (60.0)	15 (50.0)	
Left	41 (45.6)	14 (46.7)	12 (40.0)	15 (50.0)	
Dominant limb, n (%):					0.092*
Right	84 (93.3)	25 (83.3)	29 (96.7)	30 (100.0)	
Left	2 (2.2)	2 (6.67)	0 (0.0)	0 (0.0)	
Both	4 (4.4)	3 (10.0)	1 (3.3)	0 (0.0)	

*Notes:* p < 0.05 is indicated in bold, * chi-square test; ** one-way ANOVA

*Abbreviations: M* means, *SD* standard deviations, *p* statistical significance level, *n* number of participants

**Fig. 2:**
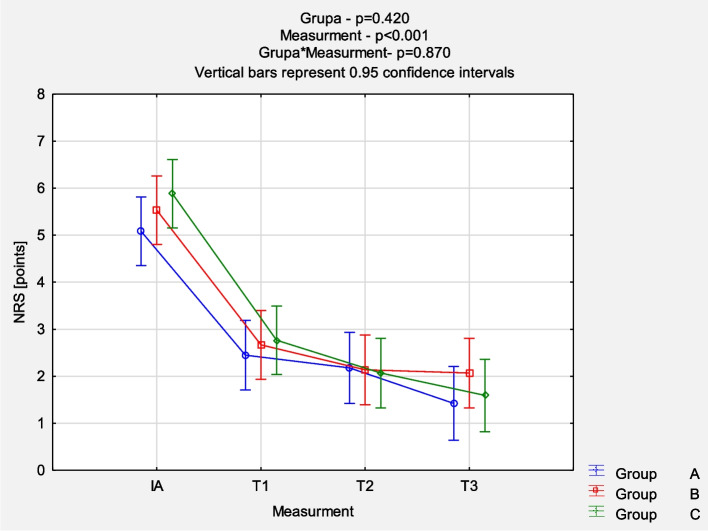
Comparison of NRS results between the groups in each measurement point. *Abbreviations: NRS* Numeric Rating Scale, *IA* initial assessment, *T1* assessment after 6 weeks, *T2* assessment after 12 weeks, *T3* assessment after 24 weeks

**Fig. 3:**
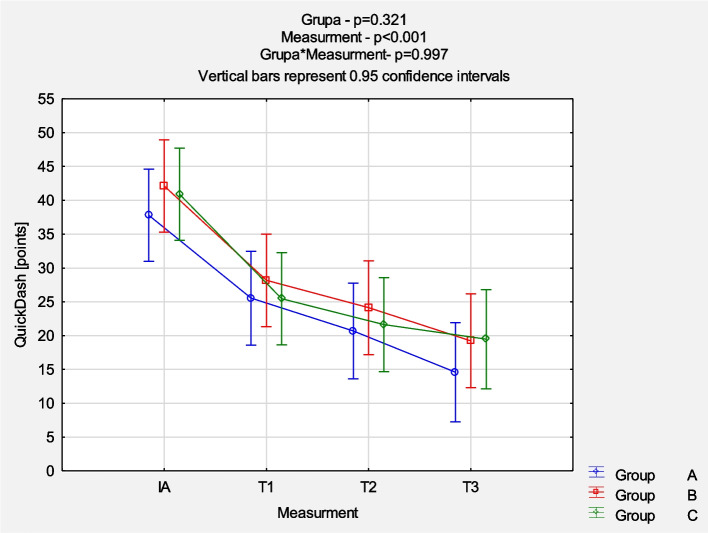
Comparison of QuickDash results between the groups in each measurement point. *Abbreviations: IA* initial assessment, *T1* assessment after 6 weeks, *T2* assessment after 12 weeks, *T3* assessment after 24 weeks

**Fig. 4:**
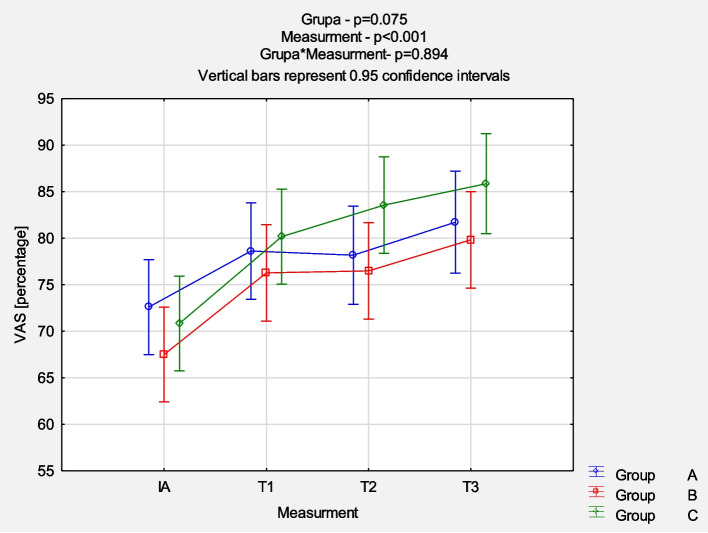
Comparison of EQ-5D-5L VAS results between the groups in each measurement point. *Abbreviations: VAS* Visual Analogue Scale (EQ-5D-5L), *IA* initial assessment, *T1* assessment after 6 weeks, *T2* assessment after 12 weeks, *T3* assessment after 24 weeks

**Fig. 5:**
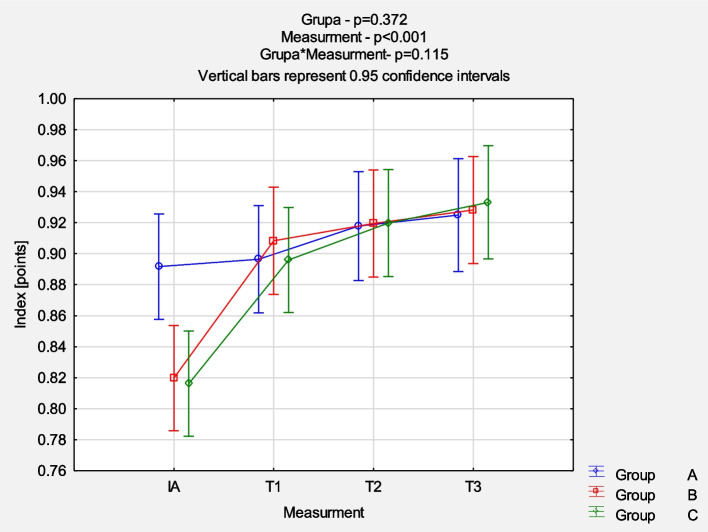
Comparison of EQ-5D-5L Index results between the groups in each measurement point. *Abbreviations: Index* utility index (EQ-5D-5L), *IA* initial assessment, *T1* assessment after 6 weeks, *T2* assessment after 12 weeks, *T3* assessment after 24 weeks

The comparison of NRS evolution between the groups revealed a reduction in pain intensity mostly in the first 6 weeks of follow-up (*p* < 0.001), but no significant statistical differences between groups were noticed (*p* = 0.870). Detailed results are presented in Fig. 2. The comparison of QuickDash results between the groups at each measurement point also showed a similar pattern of mean value reduction (*p* < 0.001) without significant statistical differences between groups (*p* = 0.997). Figure 3 presents the detailed results. The comparison of EQ-5D-5L VAS results between the groups at each measurement point demonstrated no statistically significant differences. The dynamics of changes 6 weeks after the last injection were similarly more intense (*p* < 0.001), as shown in Fig. 4. The comparison of EQ-5D-5L Index results between the groups at each measurement point demonstrated no statistically significant differences. The dynamics of changes 6 weeks after the last injection are similarly more intense (p < 0.001) (Fig. 5).

**Fig. 6:**
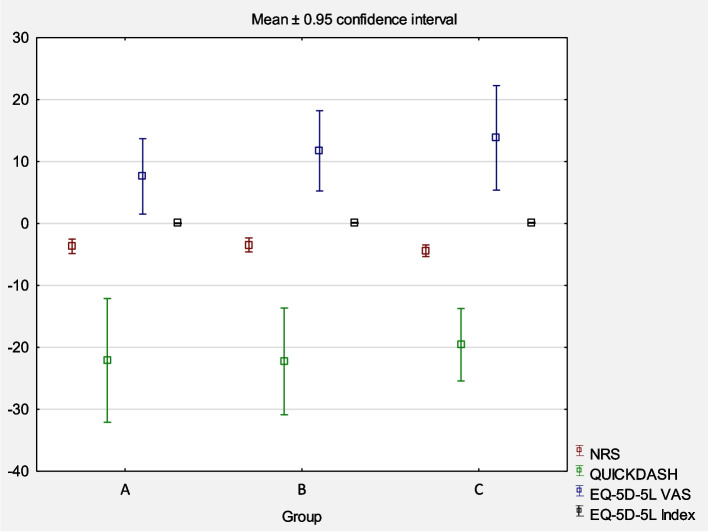
Differences in the mean initial (IA) and final values (T3) for primary outcomes in the groups. *Abbreviations: NRS* Numeric Rating Scale, *VAS* Visual Analogue Scale (EQ-5D-5L)

**Fig. 7:**
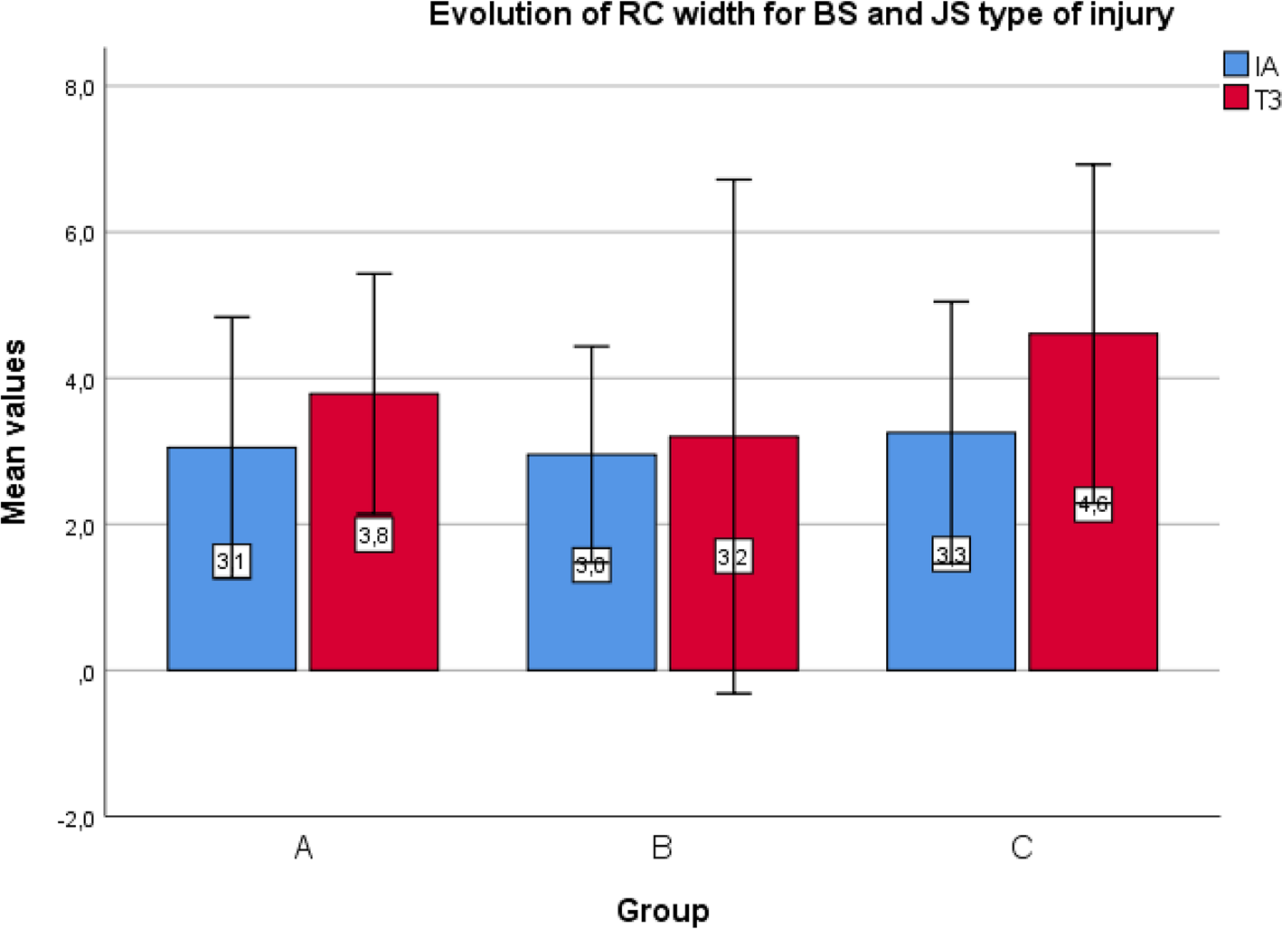
Mean increase of width for BS and JS type of injury for specific groups. *Abbreviations: RC* rotator cuff, *BS* bursa-sided, *JS* joint-sided, *IA* initial assessment, *T3* assessment after 24 weeks

**Fig. 8:**
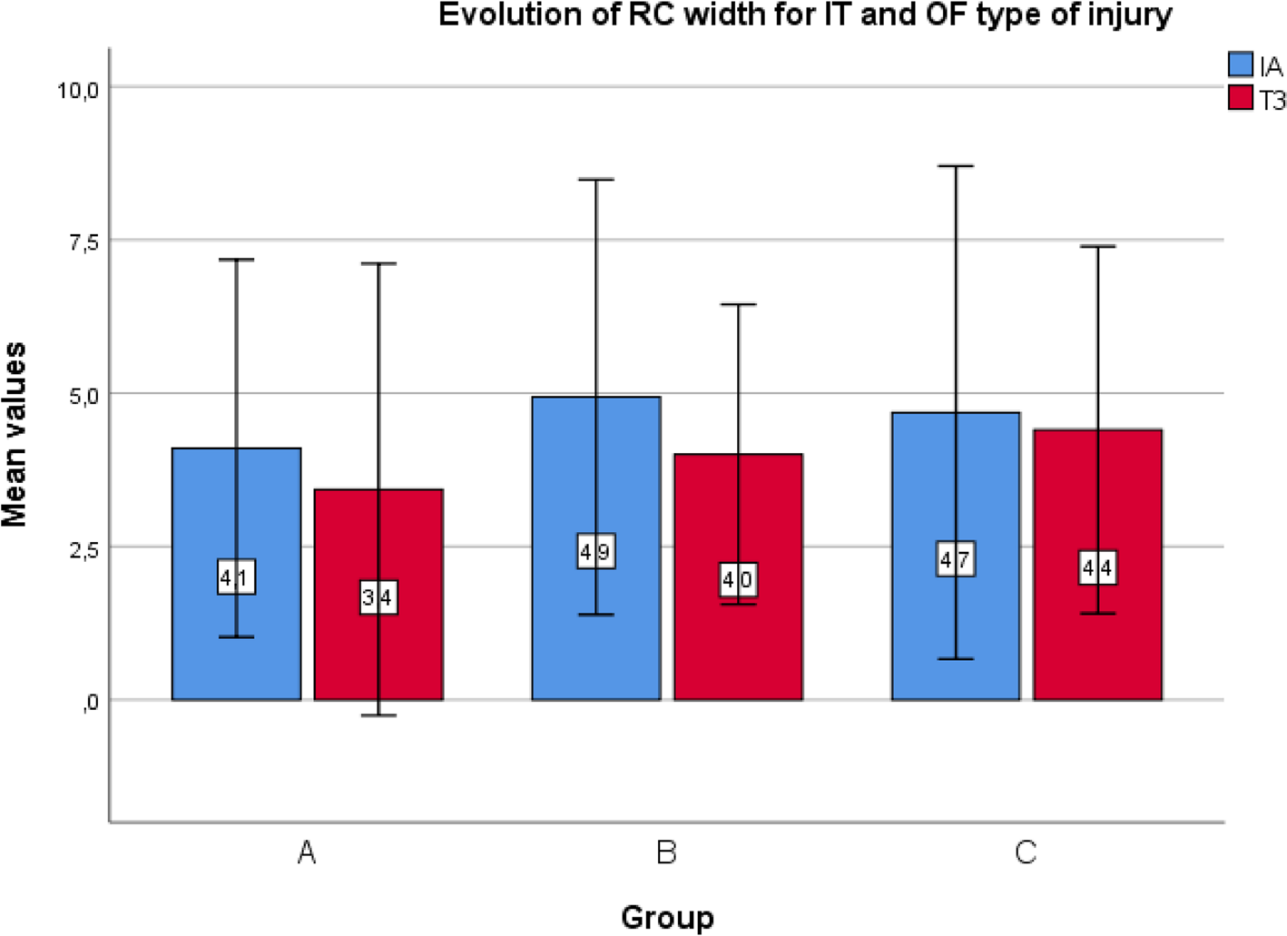
Mean reduction of width for IT and OF type of injury for specific groups. *Abbreviations: RC* rotator cuff, *IT* intra-tendon, *OF* oblique or focal, *IA* initial assessment, *T3* = assessment after 24 weeks

The differences between values at baseline IA and T3, as well as the mean value of these differences for each studied group, were calculated. Analysis of afferences in the mean initial (IA) and final values (T3) for primary outcomes between the subsequent comparative groups did not show statistical significances (*p* > 0.05). Detailed results are presented in Fig. 6. Lost continuity of RC between IA and T3 was found in three cases (one in each group), and the number of cases with RC regeneration confirmed by ultrasound was 22 in Group A, 20 in Group B, and 23 in Group C. The mean increase in RC width in BS and JS types of injury was 0.7 mm, 0.2 mm, and 1.3 mm for Groups A, B, and C, respectively. There was a statistically significant difference between Groups B and C (*p* < 0.05) (Fig. 7). The mean width reduction for IT and OF types of injury was 0.7 mm, 0.9 mm, and 0.3 mm for Groups A, B, and C, respectively. No statistically significant difference was found between the groups. No significant harms, complications, or unintended effects of the treatment were reported (*p* > 0.05) (Fig. 8).

## Discussion

Various arthroscopic techniques are used in PTRCI, but there is still limited evidence supporting the advantages of one procedure over the others in terms of clinical effectiveness and the incidence of complications [23, 24]. Fama et al. [25] noted that patients with PTRCI of the supraspinatus tendon treated with the tear completion repair procedure with removal of the “critical zone” and biological stimulation via microfractures showed good outcomes, including constant score (CS) and range of motion (ROM), pain intensity (VAS), and MRI or ultrasound examinations. Moreover, in this study, conservative treatment of PTRCI with injections of collagen and PRP as monotherapy or combined therapy showed no significant difference in efficacy.

Degenerative rotator cuff tendinopathy appearing as PTRCI is a condition that is challenging to treat, mainly because of the poor regenerative potential of tendons correlated with ageing. Many other factors contributing to treatment failure have been described, such as overload in the rehabilitation process, drugs (i.e., quinolones), alcohol intake, smoking, and corticosteroids [26]. There has been a growing interest in biologically active substances, such as growth factors, stem cells, and autologous conditioned serum [27].

There are many publications about PRP’s potential enhancement of healing potential after surgical repairing of RCI and decreasing the ratio of retear. However, the data are conflicting [28–31]. In vitro, culture experiments confirm the anabolic effect of PRP on the healing of RC lesions through cell proliferation and the synthesis of collagen I [32]. However, in vivo data, especially without RC repair, show much confusion about the benefits of PRP and the conclusions are conflicting. Freitag et al. [33] used a PRP protocol similar to our study’s and presented a case report of a 60-year-old patient treated with three doses of PRP for PTRCI at weekly intervals administered not into the bursa but into the partial supraspinatus tear using a lateral approach. The patient was followed up for 52 weeks. The NRS, patient percentage perceived improvement (PPPI), and handheld isometric dynamometer assessment of RC strength were recorded in follow-up intervals at 6, 17, 25, and 52 weeks, revealing the best PPPI up to 90% at Week 17 and slightly worse outcomes at the 52nd week (70%).

Scarpone et al. [34] presented an open study prospective trial without a control group of 19 patients treated with a single ultrasound-guided intralesional injection of PRP in RCT, reporting satisfying results in 18 cases up to 52 weeks of follow-up. Similar outcomes were achieved by Wesner et al. [35] in their pilot study on a small group of nine participants versus a control group placebo (7 with PRP and 2 with saline) in the 6-month follow-up. Kesikburun et al. [36] also used a single injection of PRP and performed RCT with a 1-year follow-up, in which PRP injections versus placebo (saline) were injected into subacromial bursa in a group of 40 patients (20 – PRP versus 20 – saline). Injection therapy was followed by a 6-week rehabilitation programme. The authors found no significant differences in improved quality of life, pain, disability, and shoulder range of motion compared to the placebo in patients with PTRCI who were treated with an exercise programme.

However, none of the above-cited studies had subsequent imaging controls to illustrate tendon regeneration. It seems reasonable to raise the question of an insufficient dose of single-shot PRP (especially if a low-volume whole blood set was used) as a possible reason for the unsatisfactory results. Similar questions have been raised concerning the type of PRP that may be optimal for promoting regeneration, as confirmed in comparative laboratory studies of low and high leucocyte PRP [17, 37]. We found only one prospective study performed by Nestorova et al. [20], in which 22 patients with PTRCI were treated with a total of 20 intrabursal collagen GUNA MD injections for 8 weeks, with satisfactory results achieved in 73% of the patients, and 77% showed recovered lesions by ultrasound.

Our study clearly showed the potential to heal injured RC, regardless of how weak, which can be activated or augmented by external delivery of active biological substrates, although without a clear difference between monotherapy and combined therapy. However, many questions remain open about the optimal PRP composition, collagen dose, administration sequence (mixture or sequential administration), and injection location, depending on the type of RC injury (intraarticular or intrabursal). The most interesting future direction seems to be elucidating the unknown connection between the structural integrity of RC and clinical outcomes.

### Strengths and limitations

The strength of our study is in being the first in vivo investigation of the potential synergy between PRP and collagen delivered into the subacromial bursa in terms of tendon regeneration by randomised control trial (the authors did not find a similar study in the literature). In addition to the well-validated subjective assessment questionnaires, we used ultrasound examinations with 6 months of observation, which seemed to be long enough to observe changes in echogenicity and possible changes in tendon thickness.

One limitation of the study is certainly the small group of participants and operator-dependent imperfections in the ultrasound methodology of RC thickness measurement. However, the bias is mainly connected to difficulties in obtaining the same cross-section point of reference for precise test–retest measurement. Another bias that may influence the results is the wide margin of tolerance allowed in the rehabilitation protocol implemented for the participants before or in the course of the study, which was beyond our control, as well as a sport or working activities exerted by many of them against recommendations. There were also no restrictions on taking painkillers when needed during the observation period.

## Conclusions

Our findings suggest that a combined therapy of collagen and PRP in PTRCI is not more effective than monotherapies in reducing pain and anxiety/depression symptoms or improving mobility, self-care, and usual activities. This pilot study will be continued with a larger group of patients, considering objective measurement tools.

## Data Availability

The datasets used and/or analysed during the current study are available from the corresponding author on reasonable request.

## Abbreviations

ACS: autologous conditioned serum
AD: anxiety and depression
BS: bursa-sided
IA: initial assessment
ISP: infraspinatus
IT: intra-tendon
JS: joint–sided
LHB: long head biceps
MRI: magnetic resonance imaging
NRS: Numeric Rating Scale
OF: oblique or focal
PD: pain and discomfort
PRP: platelet-rich plasma
PTRCI: partial-thickness rotator cuff injuries
RCI: rotator cuff injuries
RCT: rotator cuff tendinopathy
SSC: subscapularis
SSP: supraspinatus tendon
